# Supplementary material for: Human Osteoblasts’ Response to Biomaterials for Subchondral Bone Regeneration in Standard and Aggressive Environments
Source: Int J Mol Sci. 2023 Sep 29;24(19):14764. doi: 10.3390/ijms241914764 (PMC10573262; doi:10.3390/ijms241914764)
Supplement: Supplementary file 1 [file ijms-24-14764-s001.zip › ijms-2573299-supplementary.pdf]

SUPPLEMENTARY MATERIAL AND METHOS

H_2O_2 treatment conditions setting

The exposure of the cells to 400 μM H_2O_2 in absence of materials was mandatory to prove the effects of this type of stimulation, before to apply the present experimental model to the cell/scaffolds, which should mimic the altered joint microenvironment in elderly patients. Therefore, before proceeding with the experiments on biomaterials it was necessary to fine-tune some parameters, such as cell density, H_2O_2 concentration, and time of exposure. Two different cell densities ($2x10^4$ and $3x10^4$ cells/cm^2), increasing H_2O_2 concentrations (300, 400, and 500 μM), and different times of exposure (24, 48, and 72 hours) have been tested on NHOst cultured on tissue-culture polystyrene multiwells by cell viability test and gene expression analysis, as described in the Materials and Methods section.

Analysis of scaffold morphology

To observe possible effects of H_2O_2 on the scaffold morphology, the OC, OC+BMP-2, and OC+Sr-ACP cell/scaffolds constructs, untreated or exposed to H_2O_2, were subjected to paraffin embedding and, before the H&E staining, observed by optical microscope.

SUPPLEMENTARY RESULTS

Among the other conditions, the exposure of cells to 400 μM H_2O_2 for 24 hours followed by additional 48 hours culture was chosen based on the efficacy of H_2O_2 on cell viability without excessive toxicity. The exposure of cells to H_2O_2 induced a significant decrease in cell viability after treatment (Figure 1a). The expression of osteogenic genes, such as COL1A1, ALPL, and SPARC showed a significant decrease. On the contrary, COX2 showed a significant increase in treated cells, whereas iNOS was higher in treated cells although not significantly different from the control (Figure 1b).

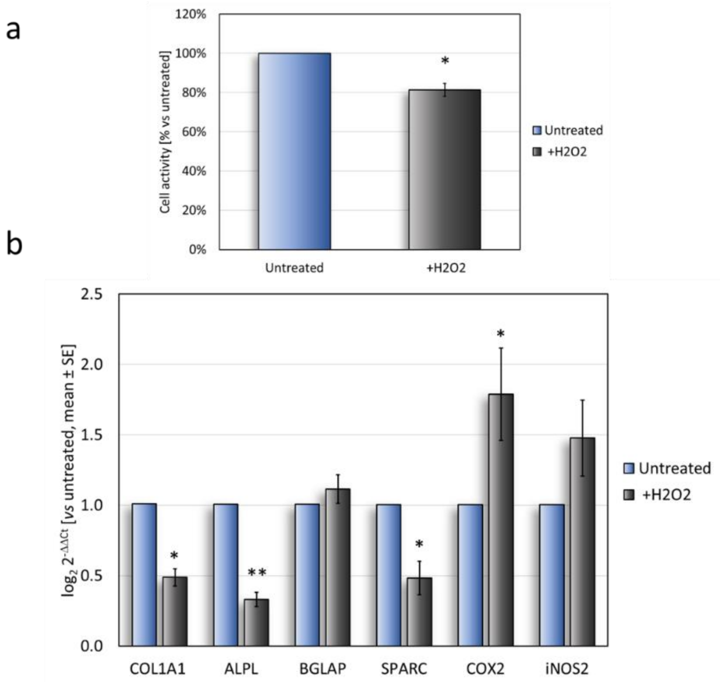

Figure S1. Effects of H_2O_2 treatment on NHOst viability and gene expression. (**a**) Cell viability reported as percentage with respect to untreated cells. (**b**) Relative gene expression of COL1A1, ALPL, BGLAP, SPARC, COX2, and iNOS2 reported as fold change with respect to untreated cells value (1). Mean ± SE, n=3 replicates. *p<0.05; ** p<0.005, untreated vs H_2O_2.

At the optical microscope observation, no appreciable differences were found between the untreated and H_2O_2 treated and OC and OC+BMP-2 unstained scaffolds. On the other side, in the OC+Sr-ACP scaffolds with large visible Sr-ACP granules, there was an evident difference between the two conditions (Figure 2).

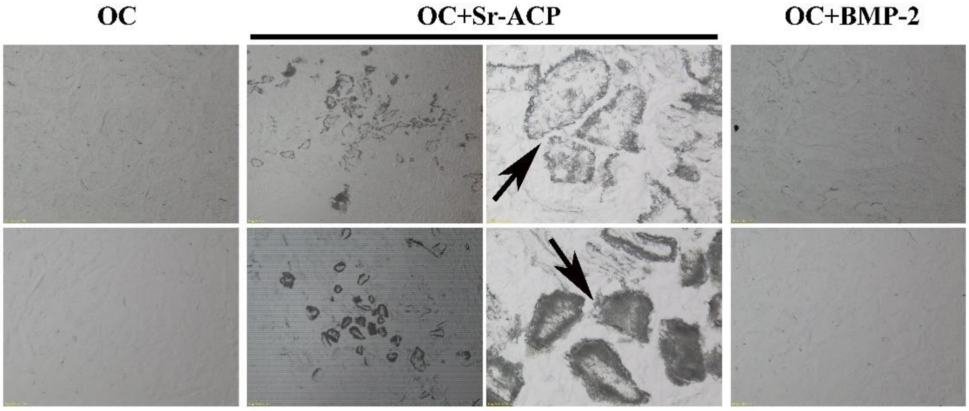

Figure S2. Scaffold morphology after H_2O_2 treatment. Representative images of the untreated (a) and H_2O_2 treated (b) scaffolds before the H&E staining. The black arrows on the far-right panels indicate Sr-ACP granules. Magnification 40x
